# Detection of a Soluble Form of CD109 in Serum of CD109 Transgenic and Tumor Xenografted Mice

**DOI:** 10.1371/journal.pone.0083385

**Published:** 2014-01-06

**Authors:** Hiroki Sakakura, Yoshiki Murakumo, Shinji Mii, Sumitaka Hagiwara, Takuya Kato, Masato Asai, Akiyoshi Hoshino, Noriyuki Yamamoto, Sayaka Sobue, Masatoshi Ichihara, Minoru Ueda, Masahide Takahashi

**Affiliations:** 1 Department of Pathology, Nagoya University Graduate School of Medicine, Nagoya, Aichi, Japan; 2 Department of Oral and Maxillofacial Surgery, Nagoya University Graduate School of Medicine, Nagoya, Aichi, Japan; 3 Department of Pathology, Kitasato University School of Medicine, Sagamihara, Kanagawa, Japan; 4 Department of Biomedical Sciences, College of Life and Health Sciences, Chubu University, Kasugai, Aichi, Japan; Institute of Cancerology Gustave Roussy, France

## Abstract

CD109, a glycosylphosphatidylinositol-anchored glycoprotein, is expressed at high levels in some human tumors including squamous cell carcinomas. As CD109 is reportedly cleaved by furin and its soluble form is secreted into culture medium *in vitro*, we hypothesized that CD109 could serve as a tumor marker *in vivo*. In this study, we investigated CD109 as a novel serum tumor marker using transgenic mice that overexpress mouse CD109 (mCD109-TG mice) and tumor xenografted mice inoculated with human CD109 (hCD109)-overexpressing HEK293 cells. In sera and urine of mCD109-TG mice, mCD109 was detected using western blotting. In xenografted mice, hCD109 secreted from inoculated tumors was detected in sera, using western blotting and CD109 ELISA. Concentrations of tumor-secreted CD109 increased proportionally as tumors enlarged. Concentrations of secreted CD109 decreased notably by 17 h after tumor resection, and became undetectable 48 h after resection. The half-life of tumor-secreted CD109 was about 5.86±0.17 h. These results indicate that CD109 is present in serum as a soluble form, and suggest its potential as a novel tumor marker in patients with cancers that express CD109.

## Introduction

CD109, a glycosylphosphatidylinositol (GPI)-anchored cell-surface glycoprotein, is a member of the α2-macroglobulin/C3,C4,C5 family [1]–[4]. CD109 was identified as a cell-surface antigen expressed in KG1a acute myeloid leukemia cells, fetal and adult CD34^+^ bone marrow mononuclear cells, activated platelets, activated T lymphoblasts, leukemic megakaryoblastes, mesenchymal stem cell subsets and endothelial cells [5]–[7]. We previously reported that, whereas CD109 is expressed in only limited cell types in normal human and mouse tissues, including myoepithelial cells of the breast, salivary, lacrimal, and bronchial secretary glands; basal cells of the prostate and bronchial epithelia; and basal to suprabasal layers of epidermis [8]–[13], its expression is frequently detected in several tumor tissues, including squamous cell carcinomas (SCCs) of the oral cavity, esophagus, lung and uterus, basal-like breast carcinoma, malignant melanoma of the skin, and urothelial carcinoma of the bladder, using immunohistochemical studies with anti-CD109 antibody [8]–[12], [14]–[16]. High expression of CD109 is also frequently detected in premalignant squamous epithelial lesions, and was associated with differentiation of SCCs in the oral cavity [14]. Reportedly, CD109 is a component of the TGF-β1 receptor system, and negatively regulates TGF-β1 signaling [17]. CD109 has been shown to be cleaved by furin into two forms, a 180-kDa soluble form and a 25-kDa membrane-attached form; the 180-kDa soluble form is secreted from the cell surface into culture medium *in vitro*. Processing CD109 into 180-kDa and 25-kDa proteins is necessary in regulating TGF-β1 signaling [18]. Moreover, release of CD109 from cell surfaces, or the addition of recombinant CD109, downregulates TGF-β1 signaling and TGF-β1 receptor expression in human keratinocytes [19]. Proteomics study indicates that CD109 is released from some tumor cell lines and related to TGF-β signaling *in vitro* [20]. Thus, if a soluble form of CD109 secreted from tumors is detectable in body fluid, it could be a novel marker for malignant and premalignant lesions.

In this study, we used transgenic mice that express exogenous CD109 and xenografted mice inoculated with HEK293 cells that overexpress CD109, and found a soluble form of serum CD109 that increases proportionally with the volume of xenografted tumors. Our findings suggest that CD109 is a potential tumor marker.

## Materials and Methods

### Ethics Statement

All animal protocols were approved by the Animal Care and Use Committee of Nagoya University Graduate School of Medicine (Approval ID number: 25004).

### Antibodies

**Figure 1:**
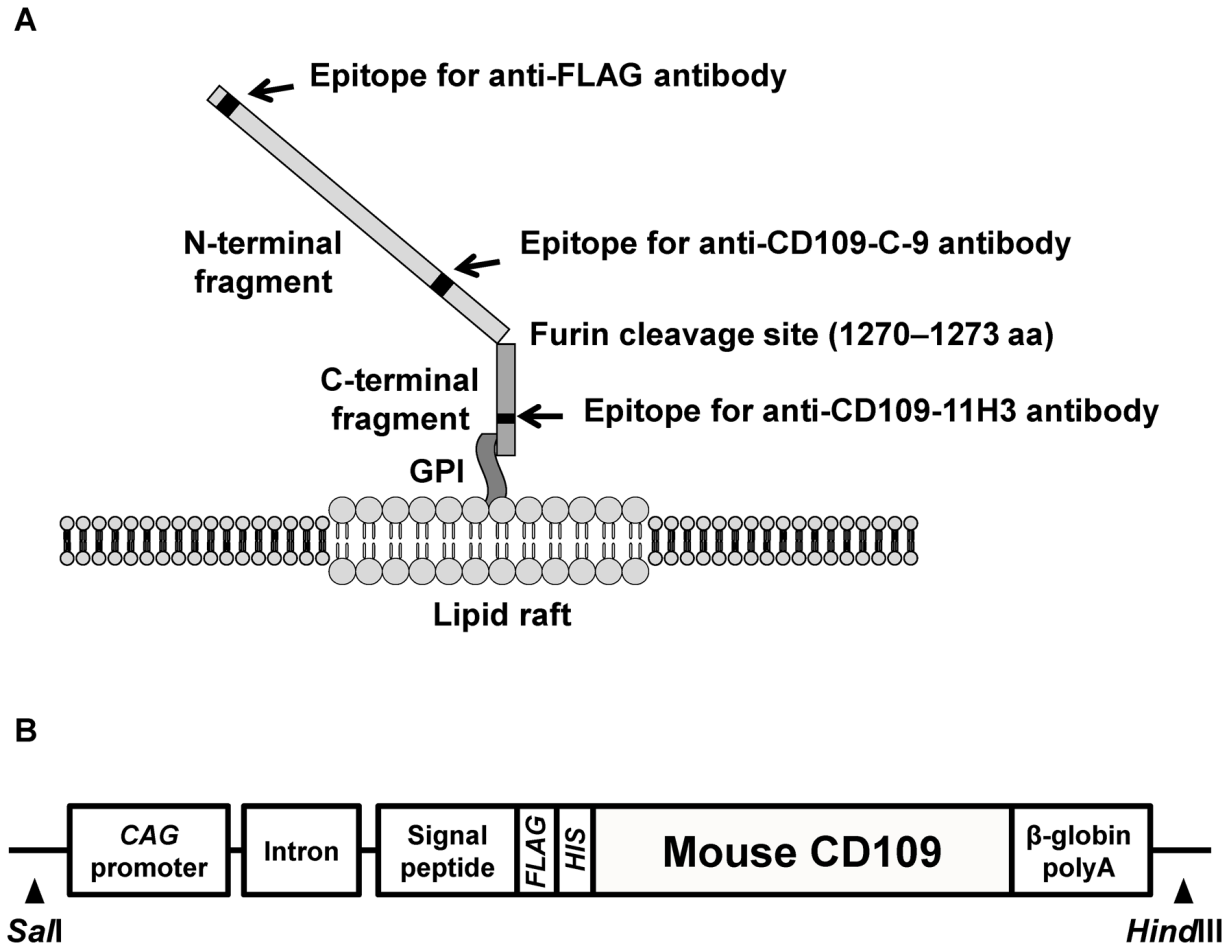
Structure of CD109 cell-surface glycoprotein. A, Schematic illustration of FLAG-tagged human CD109 (FLAG-hCD109) structure on cytoplasmic membrane. Anti-CD109-C-9 mAb, anti-FLAG mAb and anti-FLAG pAb can detect 180-kDa N-terminal fragments; anti-CD109-11H3 mAb can detect 25-kDa C-terminal fragments. B, Schematic illustration of construction of *FLAG*-tagged mouse *CD109* (*FLAG-mCD109*) transgene.

Anti-CD109-C-9 mouse monoclonal antibody (mAb), which detects 180-kDa N-terminal fragment of human and mouse CD109, was purchased from Santa Cruz Biotechnology (Santa Cruz, CA, USA; Fig. 1A). Anti-CD109-11H3 mAb, which detects 25-kDa C-terminal fragment of human CD109 was kindly provided by Immuno-Biological Laboratories Co., Ltd. (IBL, Gunma, Japan; Fig. 1A). Anti-CD109-6G1 mAb and anti-CD109-8H1 mAb, which detect 180-kDa N-terminal fragment of human CD109, but not mouse CD109, and were used for CD109 Enzyme-Linked Immunosorbent Assay (ELISA) (kindly provided by IBL). Anti-FLAG M2 mAb, anti-FLAG rabbit polyclonal antibody (pAb) and anti-β-actin mAb were purchased from Sigma (St Louis, MO, USA).

### Cells and cell culture conditions

HEK293 (derived from human embryonic kidney) cells were maintained in DMEM supplemented with 8% FBS at 37°C in 5% CO_2_ condition.

### Vector construction and generation of stable transfectants

*FLAG*-tagged human *CD109* (*FLAG-hCD109*) cDNA was cloned into pcDNA3.1(+) and used in transfection experiments as described previously [10]. Figure 1A shows a schematic illustration of FLAG-hCD109. The stable transfectants were obtained after Geneticin (Invitrogen, Carlsbad, Ca, USA) selection (HEK293-FLAG-hCD109 and HEK293-VC).

### Animals

All mice were housed in a specific pathogen-free facility. Their cages contained hardwood chip bedding at 25°C on a 12-h light/dark cycle.

### Generation of mouse *CD109* transgenic (mCD109-TG) mice

The pCAGGS vector was kindly provided by Dr. J. Miyazaki, Osaka University Graduate School of Medicine [21]. Mouse *CD109* (*mCD109*) cDNA that lack the native leader sequence was inserted into pSRαCHFX vector to generate *FLAG*- and *His*-tagged *mCD109* cDNA with sequences encoding CD8α signal peptide [8], [22]. This epitope-tagged *mCD109* was amplified by PCR with primers (forward : 5′-TTTTGGCAAAGAATTCTGCAGGCCACCATGGCCTT-3′ and reverse : 5′-CCTGAGGAGTGAATTCTCAATGTTGCACAAAGTAC-3′) and inserted between the two *Eco*RI sites of pCAGGS possessing the *CAG* promoter using In-Fusion HD Cloning Kit (Clontech, Palo Alto, CA, USA) (Fig. 1B). The construct was digested with *Sal*I and *Hin*dIII, purified and microinjected into fertilized eggs of (C57BL/6×DBA/2) F1 (BDF1) mice in Research Laboratory for Molecular Genetics of Yamagata University School of Medicine. We established three lines of mCD109-TG mice (TG-203, 206 and 213). The incorporation of the transgene was screened by PCR using primers (forward: 5′-GGCCGGATTACAAGGACGAT-3′ and reverse : 5′-GAGTGTGAGCACCCGAAACTT -3′). The transgenic mice were back-crossed for more than seven generations into the C57BL/6 strain and used for all experiments. Their nontransgenic littermates (WT-mice) were used as controls.

### Establishment of HEK293 xenografted mice

Female BALB/c-*nu*/*nu* mice were obtained from Charles River Japan (Kanagawa, Japan). Eight to 10 week-old-mice were used for HEK293 xenografted mice experiments. HEK293-FLAG-hCD109 and HEK293-VC cells were both adjusted to a concentration of 1.0×10^7^ cells suspended in 100 µl serum-free DMEM. The cell suspensions with 80 µl matrigel (Becton Dickinson, Bedford, MA, USA) were then injected subcutaneously into right flanks of BALB/c-*nu*/*nu* mice (HEK293-FLAG-hCD109, N = 7; -VC, N = 7). Tumor development was followed in individual mice every 7 days by sequential caliper measurements of length (L) and width (W). Tumor volume was calculated as volume = L×W^2^×π/6. Developed tumors were resected 42 days after xenografts. Using general anesthesia (sevoflurane) and local anesthesia (0.2% lidocaine), tumor tissue was excised with skin; wounds were then sutured. Resected tissues were cut into 5-mm^3^ specimens and quickly frozen for protein extraction or fixed in 10% neutral-buffered formalin for histological analysis.

### Preparation of serum samples

Blood from mice was collected from tail veins or retro-orbital sinuses under general anesthesia. Blood collection was performed every 7 days before tumor resection and 17, 48, 72 and 168 h after tumor resection in xenografted mice. Sera was separated by centrifugation (2000× g for 15 min) at 4°C and stored at −80°C until analysis.

### Immunoprecipitation of CD109 in the sera of xenografted mice

Sera of xenografted mice were diluted at 1∶50 in TBS buffer (50 mM Tris-HCl, 150 mM NaCl, pH 7.6). Samples were incubated for 4 h at 4°C with 20 µl Anti-FLAG M2 Affinity Gel beads (Sigma). Beads were washed 3 times with TBS buffer, bead-bound immune complexes were resuspended in 50 µl of 2× SDS sample buffer (62.5 mM Tris-HCl, pH 6.8, 2% SDS, 25% glycerol, 20 µg/ml bromophenol blue) containing 2% β-mercaptoethanol, and boiled at 100°C for 2 min. After removing beads by centrifugation, samples were subjected to western blotting.

### Western blotting

Frozen tissues were homogenized by TissueRuptor (QIAGEN, Hilden, Germany) in 5× SDS sample buffer (175 mM Tris-HCl, pH 6.8, 5% SDS, 25% glycerol, 50 µg/ml bromophenol blue) and sonicated until no longer viscous. After measuring protein concentration using the DC protein Assay Kit (Bio-Rad Laboratories, Hercules, CA, USA), lysates were boiled at 100°C for 2 min in the presence of 2% β-mercaptoethanol. Serum samples of mCD109-TG and xenografted mice were diluted at 1∶10 with PBS; equal volumes of 2× SDS sample buffer were then added to the diluted samples. They were boiled at 100°C for 2 min in the presence of 2% β-mercaptoethanol and subjected to western blotting. Urine samples were collected from mCD109-TG mice and desalted by Bio-Spin 6 column (Bio-Rad Laboratories) following manufacturer's instructions. Five×SDS sample buffer was added to the desalted samples, which were then boiled at 100°C for 2 min in the presence of 2% β-mercaptoethanol. Samples containing equal amounts of protein were applied to SDS-PAGE and transferred to polyvinylidene fluoride membranes (Millipore Corporation, Bedford, MA, USA). Membranes were blocked with Blocking One (Nacalai Tesque, Kyoto, Japan) for 1 h at RT, and then incubated with primary antibodies at 4°C overnight. After washing three times with TBST buffer (20 mM Tris-HCl, pH 7.6, 137 mM NaCl, 0.1% Tween 20), membranes were incubated with secondary antibodies conjugated to horseradish peroxidase (HRP) (Dako, Kyoto, Japan) for 1 h at RT. After washing membranes three times with TBST, antigen-antibody reaction was visualized using the ECL Detection Kit (GE Healthcare, Buckinghamshire, UK).

### Immunohistochemistry

Tumor tissues of xenografted mice were resected as described above. The tissues were fixed in 10% neutral-buffered formalin, dehydrated and embedded in paraffin. Sections (4-µm thick) were prepared for hematoxylin and eosin (HE) staining and immunohistochemistry. For immunohistochemistry, slides were deparaffinized in xylene and rehydrated in a graded ethanol series. For antigen retrieval, they were immersed into Target Retrieval Solution, pH 9.0 (Dako) and heated for 30 min at 98°C in a water bath. Non-specific binding was blocked with 10% normal goat serum for 20 min at RT. Sections were incubated with primary antibodies for 90 min at RT. Endogenous peroxidase was inhibited with 3% hydrogen peroxide in PBS for 15 min. Slides were incubated with secondary antibody conjugated to HRP-labeled polymer (EnVision+; Dako) for 25 min at RT. Reaction products were visualized with diaminobenzidine (Dako). Nuclei were counterstained with hematoxylin.

### Cell proliferation assay

Equal numbers of cells were plated in 96-well plates (2×10^3^ cells per well) with 100 µl of DMEM with 4% FBS. Cell proliferation assay was conducted 24 h after seeding using WST-1 Reagent (Roche Diagnostics, Mannheim, Germany) according to manufacturer's protocol. Absorbance was measured at 440 nm every 24 h using a PowerScan4 microplate reader (DS Pharma Medical Co. Ltd., Osaka, Japan).

### CD109 ELISA

Serum levels of CD109 from xenografted mice were assessed using the CD109 ELISA kit (kindly provided from IBL) according to manufacturer's instructions. The ELISA kit detects human CD109 but not mouse CD109. All serum samples were diluted at 1∶50 in dilution buffer; 100 µl of the individual samples and each standard were added to anti-human-CD109-6G1 mAb-coated testing plate wells and incubated at 4°C overnight. After incubation, samples were aspirated and washed 4 times with washing buffer. Then 100 µl anti-CD109-8H1 mAb conjugated to HRP-labeled solution was added and incubated for 1 h at 4°C. Wells were then washed 5 times with washing buffer, and 100 µl tetramethyl benzidine solution was added to each well and incubated for 30 min at room temperature. Finally, 100 µl stop solution was added to each well. After the reaction, absorbance of each sample was measured at 450 nm/570 nm using a PowerScan4 microplate reader (DS Pharma Medical Co.). Concentration of CD109 was calculated on the basis of a standard curve.

### Calculation of half-life of CD109 in sera of HEK293-FLAG-hCD109 xenografted mice

Half-life (T_1/2_) of CD109 in sera of HEK293-FLAG-hCD109 xenografted mice was calculated using the following equations:
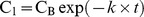


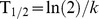
in which C_1_: concentration of CD109 at 17 h after tumor resection; C_B_: concentration of CD109 before tumor resection; *k*: rate constant of elimination; and *t*: time after tumor resection (17 h in this case) [23].

### Statistical analysis

Data are presented as mean ± SE. Statistical significance was determined with Tukey–Kramer's HSD test of one-factor factorial ANOVA using KaleidaGraph 4.0 for Windows (Synergy Software, Reading, PA, USA). *P*<0.05 was considered significant.

## Results

### CD109 is present as a soluble molecule *in vivo*

**Figure 2:**
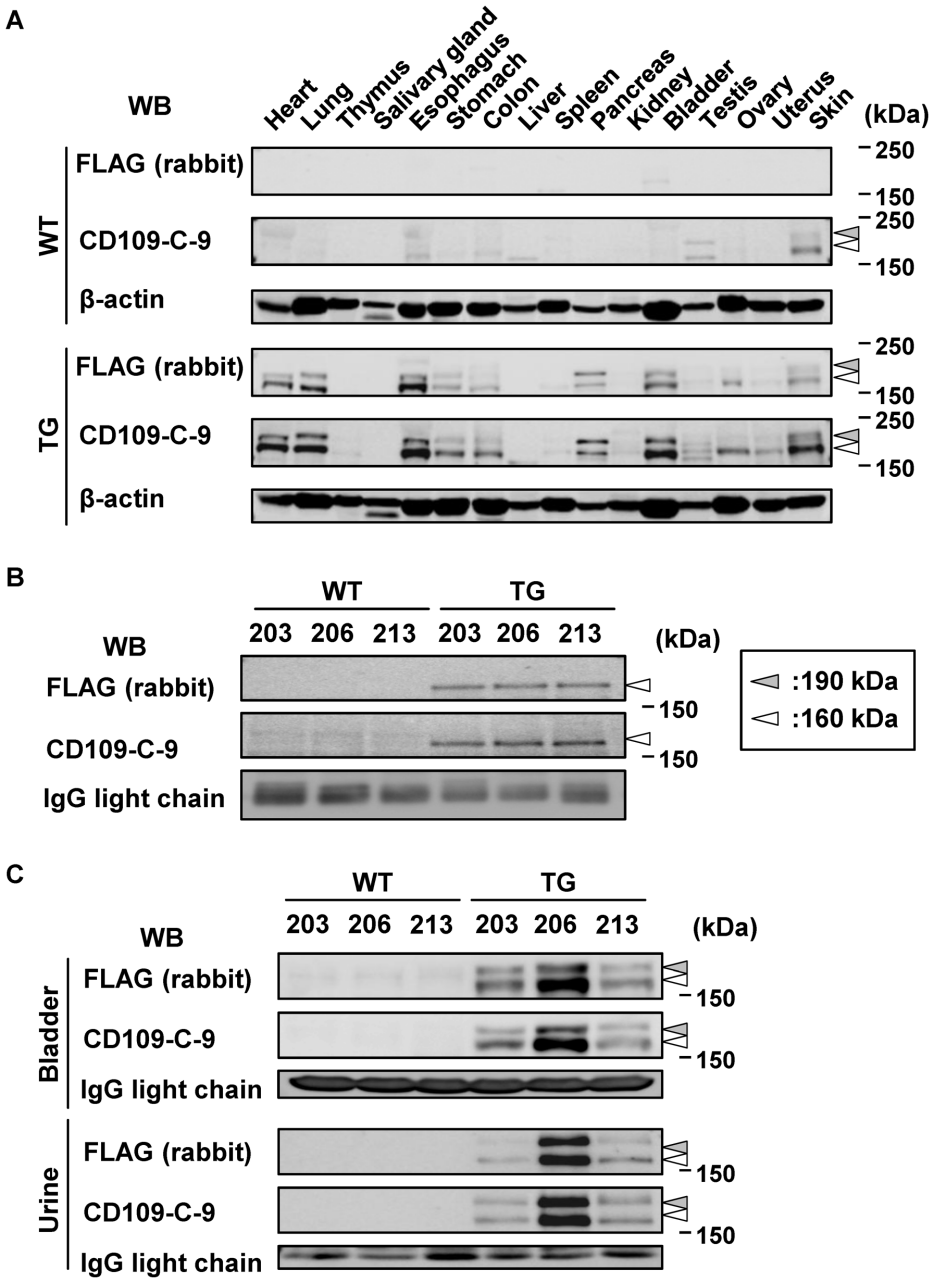
Detection of soluble CD109 in serum and urine of mCD109-TG mice. A, Expression of CD109 in various tissues of mCD109-TG (TG-206) mice. Blots of anti-β-actin antibody are shown as internal controls. White and gray arrowheads: 160- and 190-kDa bands, respectively. B, Detection of soluble CD109 in serum of mCD109-TG mice. White arrowheads:160-kDa bands. C, Expression of CD109 in bladder, and soluble CD109 in urine, of mCD109-TG mice. White and gray arrowheads:160- and 190-kDa bands, respectively. Sera, bladder tissues and urine of WT and TG siblings of 3 lines of mCD109-TG mice (TG-203, 206, and 213) were analyzed using western blots with the indicated antibodies; blots of anti-β-actin antibody and IgG light chain were used as internal controls.

After establishment of mCD109-TG mice, we assessed their tissue distribution of mCD109 expression. Whole lysates prepared from various organs of TG and WT siblings of mCD109-TG (TG-206) mice were analyzed for endogenous and exogenous mCD109 expression by western blotting with anti-FLAG pAb and anti-CD109-C-9 mAb. In lysates from a WT mouse, two bands were detected of ∼160 kDa and ∼190 kDa from testis and skin, using anti-CD109-C-9 mAb but not anti-FLAG pAb (Fig. 2A). The 160-kDa and 190-kDa bands are thought to represent N-terminal fragments of CD109 cleaved by furin and full-length CD109 before cleavage, respectively. Two bands with similar molecular masses were detected in lysates of heart, lung, esophagus, stomach, colon, spleen, pancreas, bladder, testis, ovary, uterus and skin of TG mice using both anti-CD109-C-9 mAb and anti-FLAG pAb, indicating expression of exogenous FLAG-mCD109 in mCD109-TG mice. (Fig. 2A).

We then analyzed the presence of soluble CD109 in sera and urine of TG mice, using western blotting. In serum samples from TG mice, both anti-FLAG pAb and anti-CD109-C-9 mAb recognized a major band of ∼160 kDa (Fig. 2B). However, expression of the same band was very low in samples from WT mice, as detected by anti-CD109-C-9 mAb. In TG mice urine samples, 160- and 190-kDa bands were detected with both anti-FLAG pAb and anti-CD109-C-9 mAb, and in bladder lysate, but these bands could not be detected in samples from WT mice (Fig. 2C). Both 160-kDa and 190-kDa CD109 appear to be secreted from bladder tissue. These findings indicated that CD109 is a soluble molecule in serum and urine.

### Characterization of HEK293 stable transfectants

**Figure 3:**
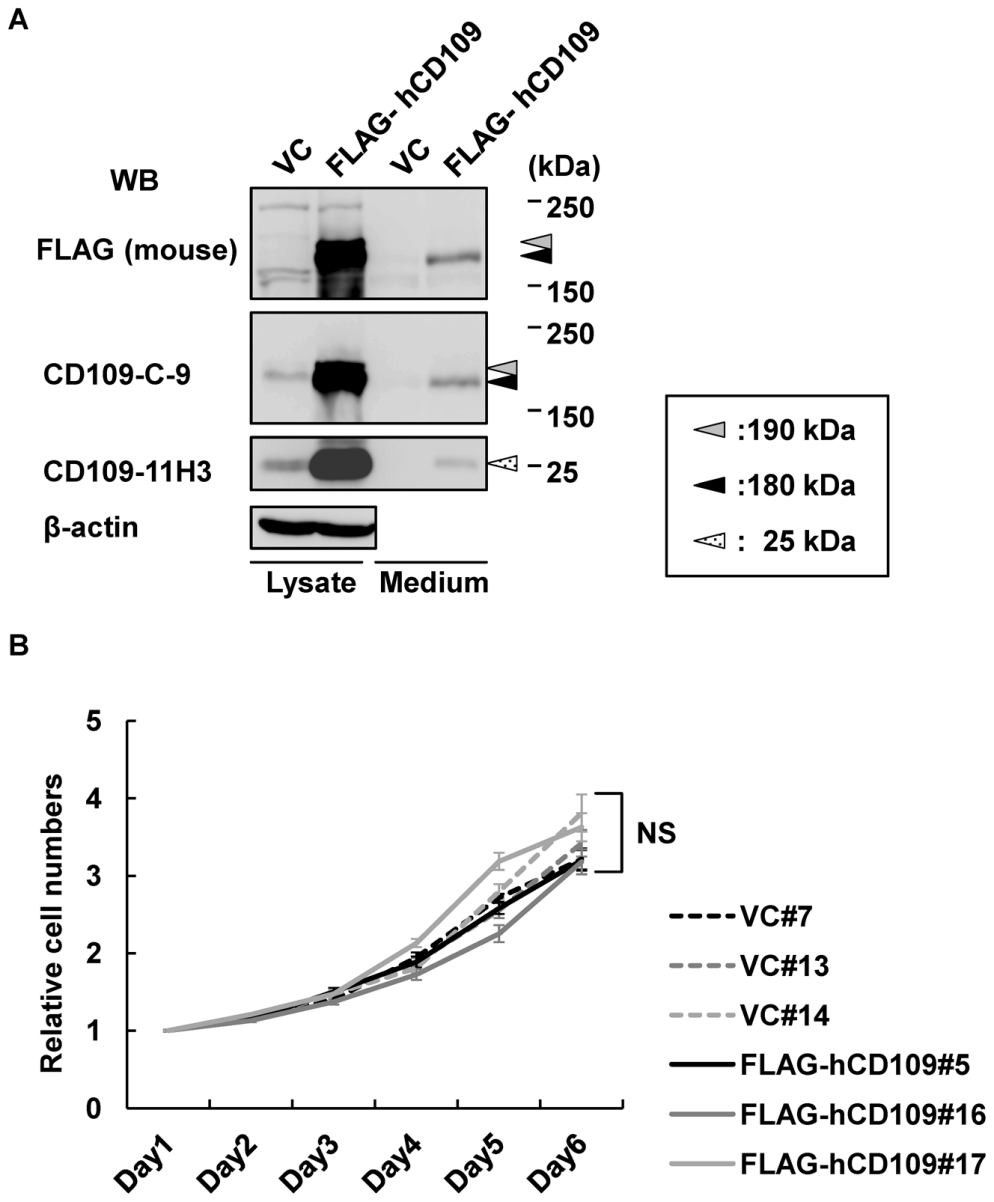
Characterization of HEK293-FLAG-hCD109 and -VC cell lines. A, Expression of CD109 in total cell lysates and detection of soluble CD109 in culture media of HEK293-FLAG-hCD109 and -VC cell line. Total cell lysates and culture media were subjected to western blotting using the indicated antibodies; blots of anti-β-actin antibody shown as internal controls. B, Cell proliferation analysis of HEK293-FLAG-hCD109 and -VC cell lines. Absorbance values at day 1 are defined as 1.0. NS: not significant, using the Tukey–Kramer's HSD test.

Before starting the xenografted mouse experiments, we characterized HEK293-FLAG-hCD109 and -VC cell lines, which were used for the xenografted mouse experiments. Expression of hCD109 in cell lysates and amount of secreted hCD109 in the culture media of these cells was assessed using western blotting with anti-FLAG, -CD109-C-9, and -CD109-11H3 antibodies. Two bands of ∼180 kDa and ∼190 kDa were detected in lysate of HEK293-FLAG-hCD109 cells using anti-FLAG and -CD109-C-9 mAbs. As previously reported, the 180-kDa band represents N-terminal fragments of CD109 cleaved by furin (Fig. 1A), and the 190-kDa band represents an immature glycosylated form of full-length CD109 [18]. The difference in molecular mass between mouse (160 kDa) and human (180 kDa) CD109 may be due to differences of glycosylation. A single 180-kDa band, which represents N-terminal fragment after the cleavage, was detected in culture medium using the same antibodies (Fig. 3A). A small amount of 180-kDa protein was also detected in cell lysate of HEK293-VC cells using anti-CD109-C-9 mAb, which was thought to be the endogenous CD109 protein. Membrane-attached 25-kDa CD109 C-terminal fragment (Fig. 1A) was detected in lysates of HEK293-FLAG-hCD109 and -VC cells using anti-CD109-11H3 mAb; interestingly, a 25-kDa CD109 fragment was also faintly detected in culture medium of HEK293-FLAG-hCD109, suggesting that small amounts of C-terminal 25-kDa CD109 fragment was released from cell surfaces to culture medium. We also evaluated cell proliferation of HEK293 transfectants (Fig. 3B). No significant difference in cell proliferation was observed between HEK293-FLAG-hCD109 and -VC cell lines.

### Establishment of HEK293-FLAG-hCD109 xenografted mice

**Figure 4:**
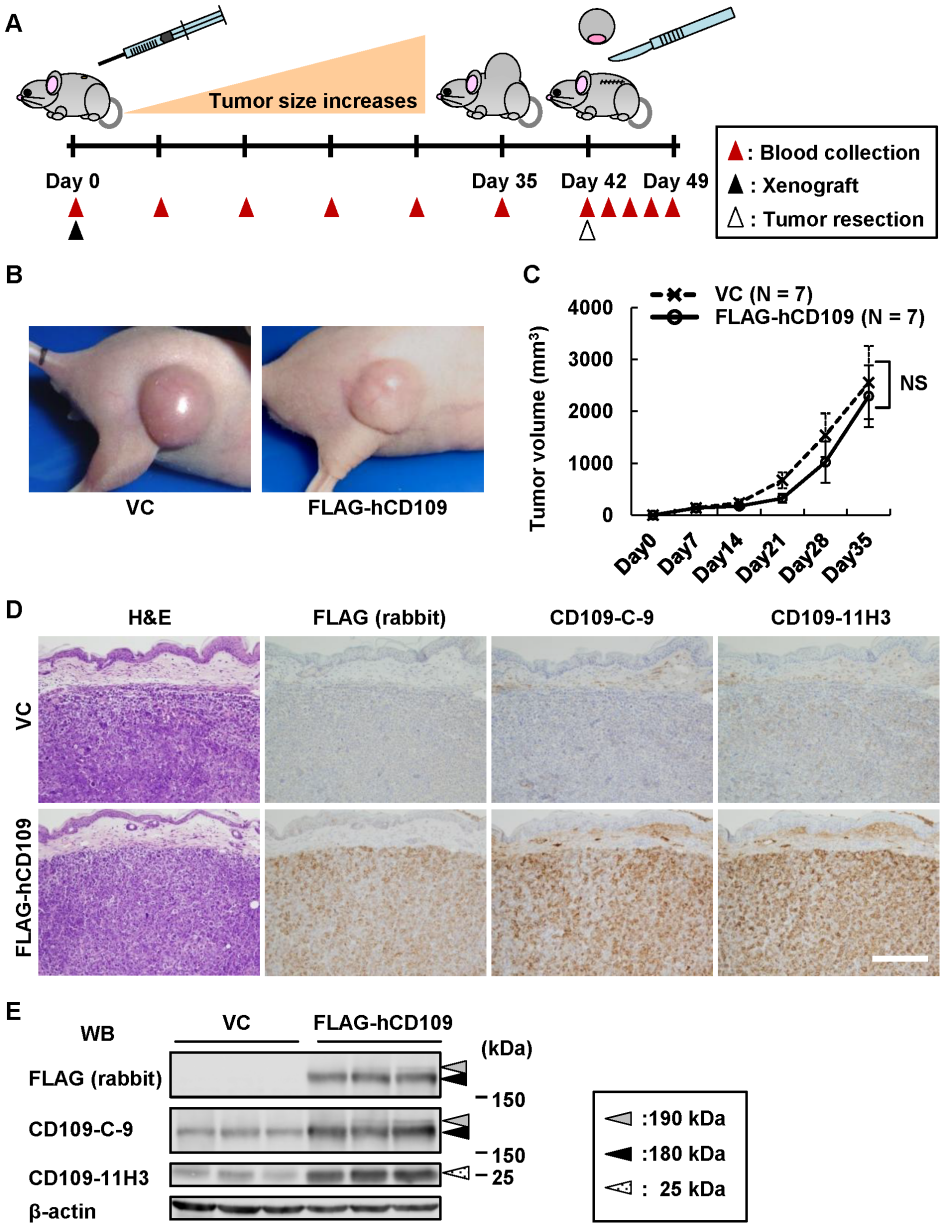
Characterization of tumors developed in xenografted mice. A, Schematic illustration of the schedule of xenografted mice experiments using HEK293-FLAG-hCD109 and -VC cell lines. Blood was collected every 7 days after the xenograft until tumor resection, and 17, 48, 72 and 168 h after tumor resection. B, Gross appearance of xenografted tumors in mice. C, Growth curves of xenografted tumors. Seven xenografted tumors of HEK293-FLAG-hCD109 and seven xenografted tumors of HEK293-VC were analyzed for tumor volume, as indicated in Materials and Methods. NS: not significant, using Tukey–Kramer's HSD test. D, Histopathological appearance of xenografted tumors. Sections of xenografted tumors resected at Day 42 were subjected to H&E staining, and immunohistochemical staining with the indicated antibodies. Scale bar: 200 µm. E, Expression of CD109 in xenografted tumors. Total cell lysates from three of each xenografted tumor groups of HEK293-FLAG-hCD109 and -VC cell lines were analyzed for CD109 expression by western blotting. Dotted-white, black and gray arrowheads: 25-, 180- and 190-kDa bands, respectively. Blots of anti-β-actin antibody shown as internal control.

To verify whether CD109 secreted from tumors is detectably present in body fluids, we established xenografted mice using HEK293-FLAG-hCD109 and -VC cells. The schedule of the xenografted mouse experiment is schematically illustrated in Figure 4A. Xenografted cells injected in BALB/c nude mice were allowed to grow for 42 days; tumors derived from the xenografted cells were resected. The developed tumors were round, movable and sharply marginated. No significant difference was found between tumors from HEK293-FLAG-hCD109 and -VC cells in gross pathological findings or growth rates (Fig. 4B,C). No apparent adhesion or invasion to adjacent tissues was observed in any tumors derived from either cell line. It is unclear why there was no difference in tumor growth between HEK293-FLAG-hCD109 and -VC xenografts, although CD109 is reportedly a negative regulator of TGF-β signaling.

The resected tumor tissue was evaluated by H&E staining and immunohistochemistry. The HEK293-FLAG-hCD109 tumors showed strong expression of exogenous FLAG-hCD109 when immunostained with anti-FLAG pAb, anti-CD109-C-9 and -CD109-11H3 mAbs. However, no histological differences between HEK293-FLAG-hCD109 and -VC tumors were observed in the H&E-stained sections (Fig. 4D). Cell lysates of tumor tissues were analyzed by western blotting with anti-FLAG pAb, anti-CD109-C-9 and -CD109-11H3 mAb, which confirmed high CD109 expression in HEK293-FLAG-hCD109 tumor tissues compared with that in HEK293-VC tumor tissues (Fig. 4E).

### Tumor-secreted CD109 is detectable in serum and associated with tumor volume in xenografted mice

**Figure 5:**
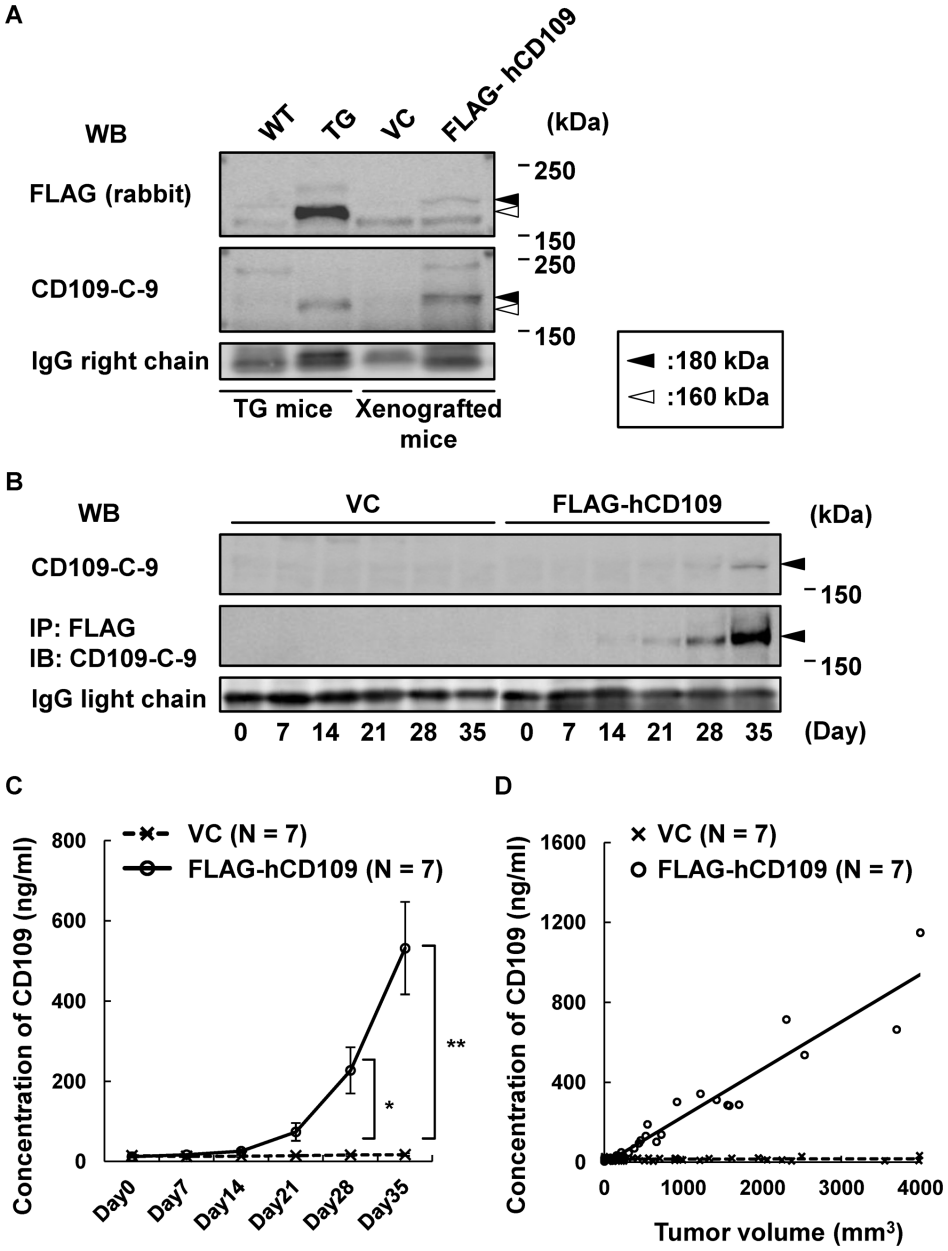
Proportional increase of soluble CD109 in serum with tumor volume in HEK293-FLAG-hCD109 xenografted mice. A, Detection of soluble CD109 in serum of HEK293-FLAG-hCD109 xenografted mice. Sera of mCD109-TG (TG-206) mice and xenografted mice were analyzed for CD109 by western blotting with the indicated antibodies. Molecular weight of mCD109: ∼160 kDa (white arrowheads); that of FLAG-hCD109: ∼180 kDa (black arrowheads); western blot for IgG light chain is indicated as an internal control. B, Analysis over time for soluble CD109 in serum of xenografted mice. Serum samples were analyzed for CD109 by western blotting with anti-CD109-C-9 mAb (upper panel). They were also analyzed for tumor-secreted FLAG-hCD109 by immunoprecipitation with Anti-FLAG M2 Affinity Gel, followed by western blotting with anti-CD109-C-9 mAb (middle panel); western blot for IgG light chain is indicated as an internal control. C, CD109 concentration in sera of xenografted mice (HEK293-FLAG-hCD109, N = 7; -VC, N = 7). Tumor-secreted FLAG-hCD109 in sera was assessed by CD109 ELISA, which recognize human CD109, but not mouse CD109. *P<0.01, **P<0.0001, using Tukey–Kramer's HSD test. D, Relationship between concentration of tumor-secreted FLAG-hCD109 in sera with tumor volumes in xenografted mice. Results in C and Fig. 4C were combined and graphically summarized.

Next, we evaluated whether FLAG-hCD109 protein released from developed tumors is detectable in serum of xenografted mice. Serum samples from xenografted mice were subjected to western blotting with anti-FLAG pAb and -CD109-C-9 mAb. Tumor-secreted FLAG-hCD109 protein in sera of xenografted mice was detected by western blotting, as was FLAG-mCD109 protein in sera of mCD109-TG mice (Fig. 5A), whereas FLAG-hCD109 could not be detected in the urine (data not shown). Tumor volume and serum CD109 concentrations were analyzed over time. Serum CD109 concentration assessed by western blotting with anti-CD109-C-9 mAb increased after Day 28, whereas assessment by western blotting combined with immunoprecipitation using Anti-FLAG M2 Affinity Gel showed increased FLAG-hCD109 after Day 14 (Fig. 5B). CD109 ELISA, which is specific for human CD109, used for quantitative estimation of serum FLAG-hCD109, detected logarithmic increase of hCD109 concentration after Day 14, when the xenografted tumors were also logarithmically growing (Figs. 4C, 5C). These results indicate that tumor-secreted CD109 in serum proportionally increases with tumor volume (Fig. 5D).

### Tumor-secreted CD109 in the serum rapidly decreases after tumor resection

**Figure 6:**
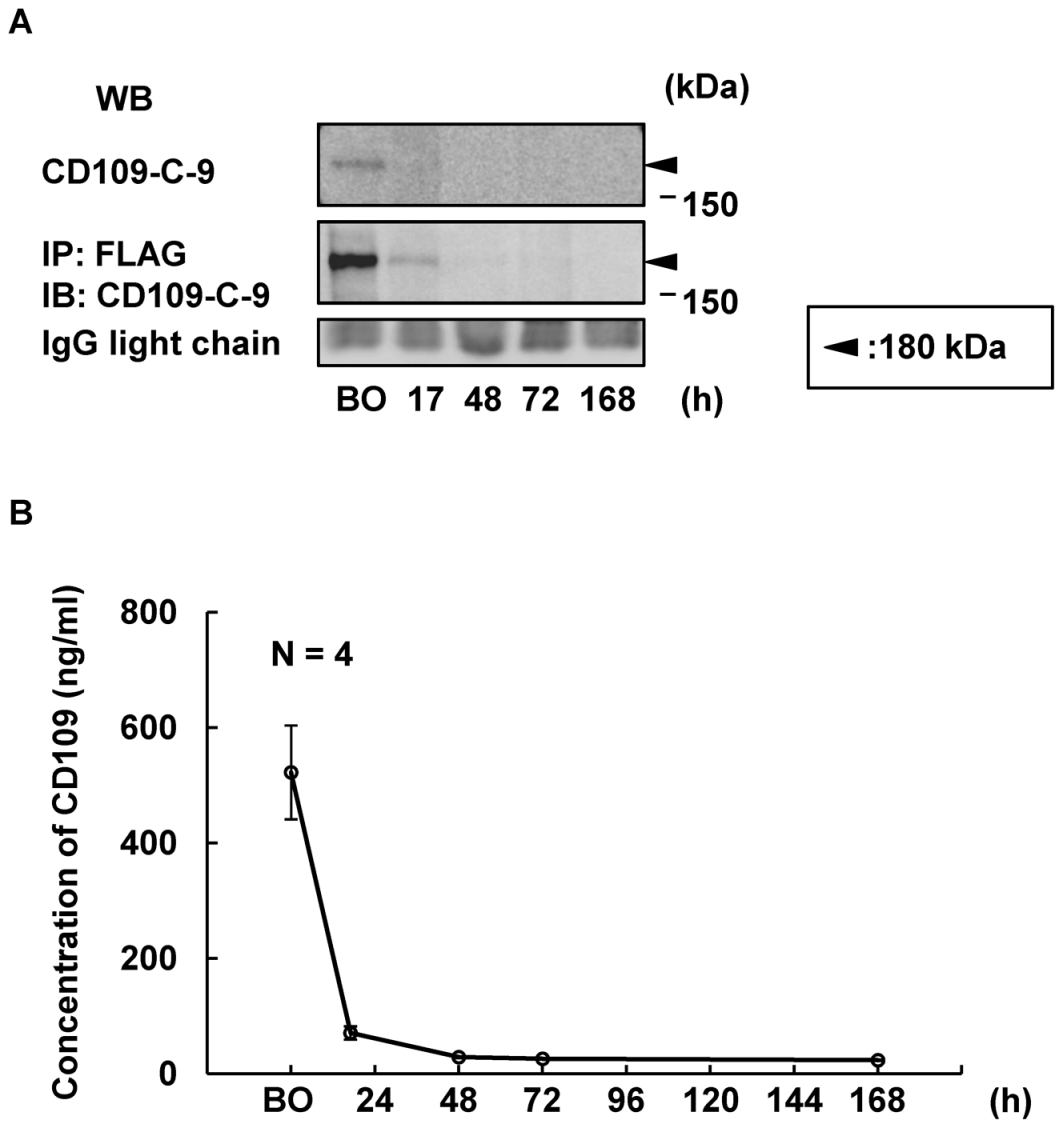
Rapid decrease of tumor-secreted FLAG-hCD109 in serum of HEK293-FLAG-hCD109 xenografted mice after tumor resection. A, Analysis over time of tumor-secreted FLAG-hCD109 in sera of HEK293-FLAG-hCD109 xenografted mice after tumor resection. Serum samples were analyzed for CD109 by western blotting with anti-CD109-C-9 mAb (upper panel). They were also analyzed for tumor-secreted FLAG-hCD109 by immunoprecipitation with Anti-FLAG M2 Affinity Gel, followed by western blotting with anti-CD109-C-9 mAb (middle panel). Black arrowheads: 180-kDa bands; western blot of IgG light chain is indicated as an internal control. B, Quantitative assessment of concentration of serum tumor-secreted FLAG-hCD109 in HEK293-FLAG-hCD109 xenografted mice after tumor resection using CD109 ELISA (N = 4). BO: Before operation.

Next, we evaluated whether tumor-secreted CD109 in the serum of HEK293-FLAG-hCD109 xenografted mice decreases after tumor resection. Forty-two days after the xenograft, tumor resections were performed as described in Materials and Methods, and serum samples were analyzed for CD109 by western blotting and ELISA. Serum CD109 concentration notably decreased 17 h after resection compared with that before resection. CD109 was undetectable 48, 72 and 168 h after the operation (Fig. 6A,B). Average concentrations of CD109 before the operation and 17 h afterwards were 521.9±81.4 ng/ml and 70.1±11.5 ng/ml, respectively. Assuming that CD109 has a monophasic elimination pattern, the half-life of tumor-secreted CD109 calculated from these results was 5.86±0.17 h, which indicates that tumor-secreted CD109 is rapidly washed out from the serum after tumor resection.

## Discussion

In the present study, we investigated the availability of CD109 as a tumor marker, using mCD109-TG mice and HEK293 xenografted mice. CD109 is highly expressed in some tumors, especially in SCCs of lung, esophagus, uterus and oral cavity, whereas it is expressed by very limited cells in normal tissues such as myoepithelial cells of the mammary, salivary and lacrimal glands, and basal cells of the prostate and bronchial epithelia. Reportedly, CD109 is cleaved and released from cell surfaces to culture media in *in vitro* studies [18]–[20]. These findings suggest the potential for CD109 in cancer management, as a novel tumor marker for SCCs or other CD109-expressing tumors. Thus, we first investigated whether the soluble form of CD109 is present in the body fluids using transgenic mice that overexpress mCD109. Our results indicate that the soluble form of CD109 is present in serum and urine of mCD109-TG mice. As CD109 was overexpressed in heart and lung of mCD109-TG mice, CD109 might be secreted by the cardiovascular system in mCD109-TG mice. Exogenous CD109 was also highly expressed in bladders of mCD109-TG mice, suggesting that soluble CD109 could be secreted from bladder epithelia. On the other hand, CD109 was not detected in urine of HEK293-FLAG-hCD109 xenografted mice (data not shown), which indicates that serum CD109 is not efficiently excreted in the urine.

In the xenografted mouse experiments, we investigated the association between tumor volume and concentration of tumor-secreted CD109 in the serum. Tumor-secreted CD109 was detected in sera of HEK293-FLAG-hCD109 xenografted mice; quantitative assessment by CD109 ELISA showed a logarithmic increase in parallel to that of xenografted tumor volume. These results suggested that serum CD109 concentration reflects amounts of CD109 secreted from tumor cells, especially from tumor tissues. CD109 is expressed in both malignant and premalignant lesions in the oral squamous epithelia. Therefore, CD109 is a new candidate among serum tumor markers for premalignant and malignant lesions. In addition, serum CD109 immediately decreased after tumor resections in this study. Seventeen hours after the operation, the concentration of CD109 was reduced to one-seventh to one-eighth, indicating that half-life of tumor-secreted CD109 is about 5.86±0.17 h. The half-life of α2-macroglobulin, a structural family protein of CD109, is reported to be several hours, which is similar to that of CD109 [24]. Compared with other tumor markers, the half-life of CD109 is longer than those of SCC antigen and CYFRA (2.2 h and 1.5 h, respectively), and is shorter than those of CEA and CA19-9 (1.5 days and 12 h, respectively) [23]. Therefore, CD109 may be a good marker for monitoring tumor progression and response to surgical treatment, which proportionally increases with tumor volume and rapidly decreases after tumor resection. On the other hand, CD109 was detected in urine of mCD109-TG mice, which exhibit high levels of exogenous FLAG-mCD109 in the bladder. CD109 did not appear to be excreted from serum to urine in the xenografted mouse experiment, suggesting that urine CD109 was secreted from bladder tissues. We previously reported that CD109 is expressed in urothelial carcinomas of the bladder in an immunohistochemical study [16]. Thus, we propose that CD109 could become a urine-based marker for urothelial carcinoma of the urinary tract.

The CD109 ELISA used in this study detects only human CD109, not mouse CD109, and clearly detected an exponential increase in tumor-produced human CD109 concentration. However, endogenous human CD109 could be present in sera of human subjects, which may confound quantitative assessments of tumor-produced CD109 in human serum, as previous publications have reported CD109 expression in activated T-cells, activated platelets, and endothelial cells; this implies that serum CD109 is secreted from hemocytes (including T-cells), platelets or endothelial cells [1]–[7]. Investigations of the utility of secretory proteins such as sIL-2, sVEGFR, sEGFR, and many other proteins as tumor markers have been widely reported [25]–[27]. However, these proteins can be difficult to use clinically because they appear in low levels in normal individuals [28]. Thus, detailed investigation of CD109 concentration in serum of normal individuals and cancer patients is required, and specific tools for detecting tumor-derived CD109 by eliminating normal tissue-derived CD109 would be quite useful for application of CD109 as a tumor marker.

Taken together, our results indicate that CD109 is a potential tumor marker. Further investigations using clinical samples from patients with carcinomas such as squamous cell carcinomas are needed to verify the usefulness of CD109 in cancer management.
